# Coverage of the Neurovascular Unit of the Fingertip Using a Reverse Homodigital Dorsal Flap

**DOI:** 10.1155/2014/315081

**Published:** 2014-01-09

**Authors:** Rosario E. Perrotta, Alessio Stivala, Dario Virzì, Roberto Grella, Domenico Pagliara, Sergio Brongo

**Affiliations:** ^1^Department of Medical and Surgery Specialties, Section of Plastic Surgery, University of Catania, Cannizzaro Hospital, 829 Via Messina, I-95126 Catania, Italy; ^2^Department of Orthopedic, Traumatologic, Rehabilitative and Plastic-Reconstructive Sciences, Second University of Naples, 3 L. De Crecchio, I-80138 Naples, Italy; ^3^Department of Medicine and Surgery, Plastic Surgery Unit, University of Salerno, Azienda Ospedaliera Universitaria OO.RR. San Giovanni di Dio e Ruggi d'Aragona, 1 Via San Leonardo, I-84131 Salerno, Italy

## Abstract

The exposure of bone, tendons, vessels, and nerves in a digital defect is one of the most frequent and severe problems to solve in hand surgery and current approaches are still disappointing. We show the use of an homodigital adipofascial flap taken from the same finger for covering the pulpar defect in a one-step surgical technique able to preserve the digital artery.

## 1. Introduction

Restoration of pulpar digital defects with exposure of bone, tendons, vessels, and nerves can be a challenging problem. Several surgical options such as local, reverse flow and free flaps can be performed [1–4]. We propose a homodigital adipofascial flap, until now used to cover only dorsal defects, in order to repair pulpar defects.

## 2. Case Presentation

**Figure 1:**
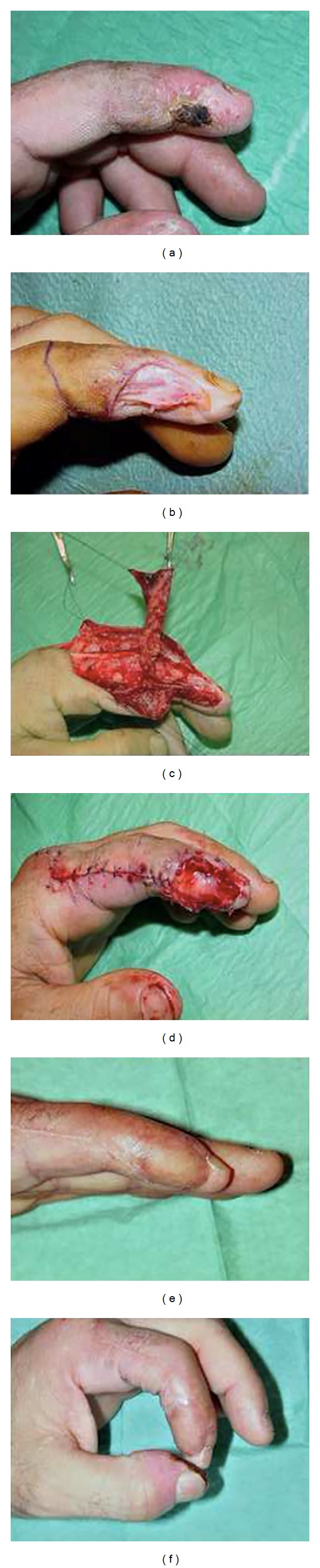
(a) Preoperative aspect of the radiodermatitis burn. (b) Exposition of the loss of substance after debridement. (c) Rotation of the adipofascial flap after dissection. (d) Closure of the wound. (e) and (f) Six-month follow-up (restoration of the clamping function of the hand).

We report a case of radiodermatitis burn that affects the clamping function of the hand. A wide pulp exposure on the terminal radial neurovascular trunk of the left index occurs as a consequence of surgical debridement (Figure 1).

The surgical procedure was carried out under locoregional anesthesia and tourniquet. Firstly, we performed a debridement, which resulted in a great exposition of terminal vessels and nerves. The “*z*” incision of the skin overlying the adipofascial flap has been created on the lateral (radial) side of the finger. Consequently, two dermoepidermal flaps were raised. In this way the adipofascial tissue was exposed and the flap was raised from proximal to distal basis. The plane of dissection was superficial to the extensor tendons sheath. During the dissection the flap was harvested very carefully, preventing trauma that might jeopardize its blood supply or devitalize the tissue. When the adequate length of dissection was completed, the tourniquet was released. Meticulous haemostasis was carried out, observing the status of blood circulation at the tip. The adipofascial flap was rotated by 180° in order to place it on the defect, and it was fixed with 5.0 absorbable suture. Over the flap a full thickness skin graft was placed. Postoperatively, the finger was kept immobilized for 72 hours, followed by gradual active mobilization. In this case we obtained the restoration of the clamping function of the hand and after a 6-month follow-up, static two-point discrimination had a mean value of 8 mm.

## 3. Discussion

**Figure 2:**
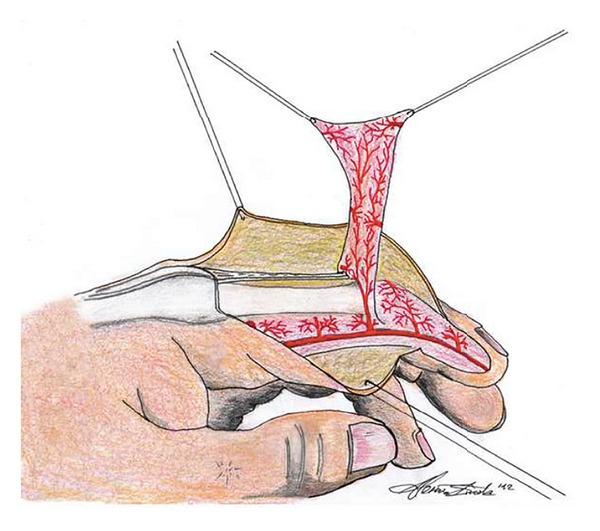
Design of the adipofascial flap describing its vascularization. The interruption of the ulnar flow allows for rotation of the flap on the defect (located at the radial side), maintaining its survival.

This technique showed several advantages. Firstly, the same finger is used to repair the loss of substance without unaesthetic defects. It is a one-stage surgical technique which allows not difficult dissection, avoiding microscopic techniques. No laterodigital arteries (main branches) are sacrificed; the adipofascial flap has a peculiar vascularization based on the dorsal communicating branches of the volar laterodigital arteries. In fact, the volar laterodigital arteries on both radial and ulnar side of the finger provide collateral dorsal communicating branches at the proximal interphalangeal joint which are numerous and constant. According to this observation, we have the chance of interrupting the flow at the ulnar side, rotating the flap on the defect (located at the radial side) and maintaining its reliability (Figure 2). Moreover, the postoperative care is simple, without immobilization of unaffected fingers and complete rehabilitation does not last long (14 days versus at least 1 month adopting other techniques). Therefore, this flap can be considered as a good alternative to reconstruction of distal pulpar defects.
